# Diagnostic yield of long-read sequencing for rare diseases: a systematic review

**DOI:** 10.3389/fgene.2026.1809097

**Published:** 2026-06-05

**Authors:** Amal Abdulsalam Ibrahim, Khalid A. Fakhro, Atiyeh M. Abdallah

**Affiliations:** 1 Department of Biomedical Sciences, College of Health Sciences, QU Health, Qatar University, Doha, Qatar; 2 College of Health and Life Sciences, Hamad Bin Khalifa University, Qatar Foundation, Doha, Qatar; 3 Laboratory of Genomic Medicine, Sidra Medicine, Doha, Qatar; 4 Department of Genetic Medicine, Weill Cornell Medicine, Doha, Qatar

**Keywords:** epigenesis, genetic, genetic diseases, genetic variation, high-throughput nucleotide sequencing, inborn, whole genome sequencing

## Abstract

**Background:**

Nearly half of patients with rare genetic disorders remain undiagnosed, which may in part be due to limitations of current short-read sequencing (SRS) approaches in detecting complex genomic alterations. Long-read whole genome sequencing (lrWGS) technologies can address these limitations through enhanced detection of structural variants (SVs), repetitive regions, and epigenetic changes.

**Methods:**

To evaluate the diagnostic yield of lrWGS in patients with rare genetic diseases receiving inconclusive or negative results from standard testing, we searched the PubMed, Science Direct, Scopus, and ProQuest databases to July 2025 for studies applying lrWGS to unresolved rare disease cases and reporting diagnostic outcomes. Risk of bias was assessed using the QUADAS-2 tool.

**Results:**

Nine studies involving 646 previously unresolved cases that underwent lrWGS met the inclusion criteria. Of these, 29 individuals (24 unique diagnoses involving 25 genes) received a definitive diagnosis through lrWGS, a diagnostic yield of 4.5%. SVs accounted for the majority of identified variants (41.67%), followed by combined SV/single-nucleotide variants (20.83%), methylation changes (16.67%), and other variant types (copy number variations, indels, and tandem repeats). Most detected variants were in regions typically inaccessible to short-read whole-exome sequencing (WES). lrWGS also enabled phasing and methylation analysis in a single assay, which was valuable for compound-heterozygosity detection and diagnostic interpretation.

**Conclusion:**

lrWGS shows clear potential for improving diagnostic rates in previously unresolved rare disease cases, particularly when applied after WES and combined with advanced tools such as phasing and methylation profiling. As technologies evolve and become more accessible, lrWGS may increasingly become a first-tier diagnostic approach, especially in phenotypically complex conditions.

**Systematic Review Registration:**

https://osf.io/y5azb/overview, identifier 10.17605/OSF.IO/Y5AZB.

## 1 Introduction

Rare genetic disorders, while individually uncommon, represent a significant global health burden in aggregate, affecting an estimated 400 million individuals worldwide, the majority (50%–75%) of whom are children (Rahit and Tarailo-Graovac, 2020). An estimated 7,000 rare disorders have been identified, with around 80% believed to have a genetic origin (Wright et al., 2018). Most of these rare genetic disorders follow Mendelian patterns of inheritance, i.e., caused by mutations in a single gene (monogenic) inherited through autosomal or sex chromosomes in a dominant or recessive manner (Chakravarti, 2021; Rahit and Tarailo-Graovac, 2020). Additionally, some rare genetic disorders arise from *de novo* (newly arising) mutations, which occur spontaneously and are not inherited from parents (Mani, 2017).

Given the predominant genetic contribution to rare diseases, next-generation sequencing (NGS) has become an essential tool for establishing a molecular diagnosis. NGS approaches include targeted or candidate gene sequencing (CGS), whole-exome sequencing (WES), whole-genome sequencing (WGS), transcriptome sequencing (RNA-seq), and methylation sequencing (MeS). Among these, WES is the most widely used and cost-effective method in clinical practice, as it focuses on protein-coding regions, which represent only 1%–2% of the genome but include approximately 85% of known pathogenic variants (Caspar et al., 2018; Sullivan et al., 2023). In cases where a patient’s phenotype strongly suggests a specific genetic disorder, targeted sequencing or gene panels can be used as a first-line diagnostic approach (Mitsuhashi and Matsumoto, 2020). However, WES has some limitations, particularly in detecting variants located outside coding regions or those involving structural variants (SVs). As a result, WGS is used in WES-negative cases to capture the whole genome and thus analyze regulatory elements, intergenic regions, intronic regions, and complex SVs that may be missed by WES (Yadav et al., 2023). The choice between WES and WGS depends on specific clinical and research objectives, yet, despite the adoption of NGS, diagnostic yield remains around 25%–50%, leaving under half of individuals with suspected Mendelian disorders undiagnosed (Mastrorosa et al., 2023; Yu et al., 2023).

One possible contributor to this diagnostic gap is the reliance of NGS on short-read sequencing (SRS), which generates reads of 50–300 base pairs, making accurate mapping and assembly difficult. This limitation leads to challenges in resolving highly repetitive sequences, GC-rich regions (e.g., 5′- and 3′-untranslated regions [UTRs]), and distinguishing functional genes from their pseudogenes. Furthermore, SRS lacks sensitivity in detecting complex SVs, such as inversions, translocations or copy number changes in repetitive regions (Dierckxsens et al., 2021; Sathyanarayana et al., 2022; Yu et al., 2023). Given that SVs play a significant role in many genetic diseases, overcoming these limitations is important for improving rare disease diagnostics (Weischenfeldt et al., 2013).

The limitations of SRS highlight the need for alternative sequencing technologies that address these limitations, such as long-read sequencing (LRS), also known as third-generation sequencing. Long-read sequencing generates reads with an average read length of 10–15 kb, enabling more accurate genome assembly and variant detection (Olivucci et al., 2024). Currently, two LRS technologies are widely available: Pacific Biosciences (PacBio) single-molecule real-time (SMRT) sequencing and Oxford Nanopore Technologies (ONT) sequencing. These platforms differ in their sequencing chemistry, data production, and analysis algorithms. PacBio uses fluorescence-based detection, where DNA polymerase incorporates labeled nucleotides, resulting in high-accuracy reads (99.9%), making it suitable for applications requiring high precision, such as rare variant detection, resolving complex genomic regions, and phasing. In contrast, ONT relies on DNA molecules passing through biological nanopores, with electrical current changes used to identify nucleotides (Zhong et al., 2021). This allows ONT to provide real-time sequencing and analysis, making it particularly useful for rapid pathogen detection, outbreak surveillance (e.g., COVID-19), and clinical microbiology where immediate results are necessary. Additionally, ONT’s portable devices, such as the MinION, enable sequencing in field or point-of-care settings, expanding its use beyond traditional laboratory environments (Zheng et al., 2023). This review does not aim to compare the two existing LRS technologies. Therefore, no further distinctions are made between them, and the two are collectively referred to as LRS throughout.

Both technologies share several key advantages over SRS, making them valuable for different clinical and research applications. Among these, a primary advantage is the ability to detect large and complex SVs that are often missed by SRS. These include large deletions, such as those affecting *PRKAR1A* in Carney complex (Merker et al., 2018) and *G6PC* in glycogen storage disease IA (Miao et al., 2018), as well as chromosomal translocations linked to developmental disorders (Dutta et al., 2019). Additionally, LRS excels in sequencing long tandem repeat expansions and GC-rich regions, which are implicated in diseases like Fragile X syndrome, myotonic dystrophy type 1, and Huntington’s disease (Ardui et al., 2017; Cummings et al., 2017; Hoijer et al., 2018; Loomis et al., 2013). Another critical advantage is its ability to differentiate clinically relevant genes from their pseudogenes, as seen in autosomal dominant polycystic kidney disease (ADPKD), enabling the differentiation of *PKD1* from highly similar pseudogenes (Borras et al., 2017). Also, LRS enables haplotype phasing, allowing the assignment of variants to maternal or paternal chromosomes, which is particularly useful when parental testing is unavailable, as demonstrated in a case where LRS confirmed the presence of two *USH2A* variants in *trans*, confirming compound heterozygosity (Gupta et al., 2023). Beyond variant detection, LRS technologies provide additional benefits. In particular, unlike PCR-based methods, LRS enables native DNA sequencing, allowing the simultaneous detection of sequence variation and methylation modifications within a single assay, without additional processing steps (Olivucci et al., 2024). These features suggest that LRS may contribute to improved diagnostic rates in Mendelian disorders, particularly in cases involving complex variants that are difficult to resolve using short-read sequencing approaches.

This systematic review aims to provide an overview of the diagnostic yield of LRS in rare disease cohorts previously undiagnosed by SRS technologies. Many other studies have reported the clinical applicability of LRS for resolving individual rare disease cases and have highlighted its advantages; others have also compared ONT and PacBio technologies (Mantere et al., 2019; Marwaha et al., 2022; Mitsuhashi and Matsumoto, 2020; Oehler et al., 2023; van Dijk et al., 2023; Yu et al., 2023). However, to our knowledge, this is the first systematic review that comprehensively assesses the diagnostic utility of LRS across rare disease cohorts, offering a broader perspective on its clinical utility.

## 2 Methods

### 2.1 Search strategy

This systematic review was conducted following the Preferred Reporting Items for Systematic Reviews and Meta-Analyses Protocols (PRISMA) guidelines (Moher et al., 2015). A comprehensive literature search was performed using the PubMed, Science Direct, Scopus, and ProQuest databases, covering studies from their inception until July 2025. The primary outcome of this review was to assess the diagnostic application of whole-genome long-read sequencing (lrWGS) technologies for rare genetic disorders, particularly in cases with previously negative, unresolved, or inconclusive genetic testing. This included evaluating the ability of lrWGS to detect single nucleotide variants (SNVs), SVs, copy number variants (CNVs), tandem repeats, and methylation patterns. The secondary outcome was to assess the types of variants (e.g., SNVs, SVs, tandem repeats) and the genomic regions involved (e.g., exonic, intronic, regulatory) among findings identified exclusively by LRS. The inclusion and exclusion criteria are summarized in Table 1. To ensure broad inclusion, the following search strategy was used: (“long read sequencing” OR “PacBio” OR “Oxford Nanopore”) AND (“Diagnosis”). Following initial screening of titles and abstracts, eligible studies were reviewed in full to confirm their inclusion in the analysis.

**TABLE 1:** Inclusion and exclusion criteria according to the PICOS statement.

Criteria	Included	Excluded
Population	Patients with undiagnosed rare genetic disorders who have negative, unresolved, or inconclusive findings from prior genetic testing	Patients with non-rare genetic disorders (such as cancer, infectious diseases, common disorders) or a diagnosed rare disorder with a known causative gene
Interventions	Studies utilizing whole genome long-read sequencing technologies (PacBio or Oxford Nanopore)	Studies utilizing targeted approaches (incl. long-reads) or other sequencing technologies
Comparators	Studies comparing whole genome long-read sequencing to standard diagnostic technologies	NA
Study design	Prospective and retrospective cohort studies, research articles	Reviews, case reports, books, protocols, guidelines, poster presentations, and animal studies
Primary outcome	Diagnostic yield of long-read sequencing in detecting a genetic cause (incl. single nucleotide variants (SNVs), structural variants (SVs), copy number variants (CNVs), tandem repeats, and methylation patterns)	NA
Secondary outcome	Variant types and genomic regions identified by LRS	NA

PICOS: population, Intervention, Comparator, Outcomes, and Study design. SNVs: single-nucleotide variants. SVs: structural variants. CNVs: copy number variants.

### 2.2 Study selection

**FIGURE 1:**
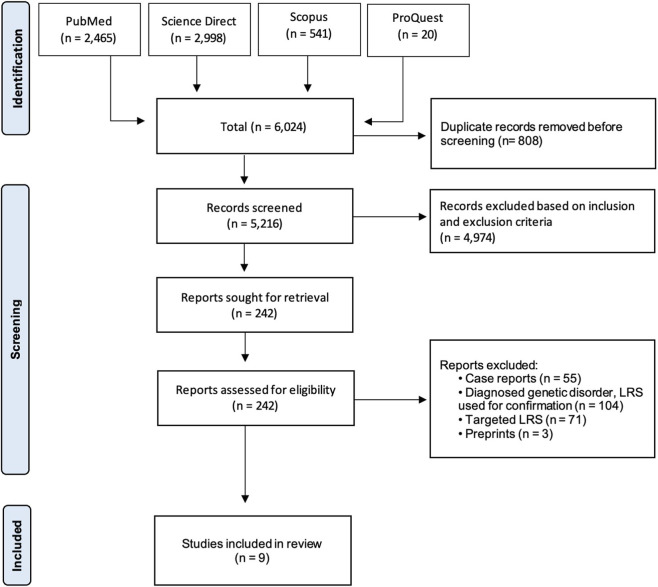
A PRISMA flow chart detailing the search strategy and study selection process.

Retrieved records were initially assessed by two authors and subsequently reviewed by the third author. Any disagreements were resolved through discussion and consensus. The total number of records retrieved from each database was documented. Studies were selected for full-text review if they met the following inclusion criteria: 1) peer-reviewed original research articles; 2) patients with undiagnosed rare genetic disorders who had previously undergone genetic testing with negative, unresolved, or inconclusive findings; 3) use of lrWGS technologies (Oxford Nanopore or PacBio); and 4) reporting of diagnostic outcomes or variant detection using long-read approaches. Studies were excluded if they met the following criteria: 1) did not utilize lrWGS; 2) focused on non-rare genetic disorders (such as cancer, infectious diseases, or other common genetic conditions), or involved a diagnosed genetic disorder with a clear phenotypic presentation and known causative gene; and 3) were review articles, case reports, conference abstracts, guidelines, animal studies, or other non-peer-reviewed material. In addition to removing duplicates, titles and abstracts were screened to eliminate irrelevant records. The remaining articles were reviewed in full to determine final eligibility. The inclusion and exclusion criteria are detailed in Table 1, and the overall screening and selection process is presented in the PRISMA flow diagram (Figure 1).

### 2.3 Quality control assessment and data extraction

The quality of eligible studies was assessed by two authors using the Quality Assessment of Diagnostic Accuracy Studies-2 (QUADAS-2) tool and reviewed by the third author (Qu et al., 2018). This tool evaluates four domains: patient selection, index test, reference standard, and flow and timing. Each domain was rated as having “low,” “high,” or “unclear” risk of bias, and any disagreements were resolved in consultation with the senior reviewers. In this review, the index test domain was frequently rated as “high” or “unclear” risk due to the lack of blinding to previous genetic test results. This limitation is expected in studies using lrWGS as a second-tier test in clinical practice. Data were extracted from the tables and full texts of all included articles and collated in a Microsoft Excel spreadsheet. The primary author performed data extraction, which was then independently reviewed by the senior authors for accuracy. Extracted variables included study design, sample size, sequencing platform (Oxford Nanopore or PacBio), diagnostic yield, and the type and complexity of detected variants (e.g., SNVs, SVs, CNVs, tandem repeats, and methylation). Where applicable, comparisons with short-read technologies were noted, as well as clinical information such as zygosity and phenotypic findings. The identified variants were confirmed using public databases, including OMIM and the UCSC Genome Browser, to check the associated phenotype, chromosomal location, and predicted variant effect.

## 3 Results

### 3.1 Search outcome

The initial search retrieved 6,024 records across four databases. After removing 808 duplicates, 5,216 records were screened by title and abstract using predefined inclusion and exclusion criteria. Of these, 4,974 records were excluded for not meeting eligibility criteria. A total of 242 full-text articles were assessed for eligibility. As a result, nine studies were included in the final analysis. The study selection process is illustrated in the PRISMA flow diagram (Figure 1), and an overview of the included studies is provided in Table 2.

**TABLE 2:** Characteristics of included studies.

Study	Country	Hospital/Data collection program	Total cases	Negative/Inconclusive cases	Cases genotyped with lrWGS	Positive control cases	Clinical focus/Disease category	Age group	Sex distribution (M:F)
Cohen et al. (2022)	United States	Children’s Mercy Research Institute in Kansas City- GA4K	1,083	584	472	125	Diverse rare diseases	P	488:595
Colin et al. (2023)	France, Belgium	AnDDI-Rares network	15	15	2	NA	Mendelian disorders	P and A	3:12
De Clercq et al. (2024)	Belgium	Ghent University Hospital	6	6	6	NA	Rare diseases with unresolved SVs	P and A	2:4
LaFlamme et al. (2024)	United States of America, Canada, Australia, New Zealand	St. Jude Children’s Research Hospital (United States of America), London Health Science Centre (Canada), Seattle Children’s Hospital (United States of America), Austin Health (Australia), University of Otago (New Zealand)	1,194	582	3	NA	DEEs	P	556:638
Sanford Kobayashi et al. (2022)	United States of America	RCIGM	35	30	35	5	Severe, syndromic pediatric disease phenotypes	P	NA
AlAbdi et al. (2023)	Saudi Arabia	KFSHRC	34 families	34 families	34 families	NA	Autosomal recessive diseases	NA	NA
Negi et al. (2025)	United States of America	Broad Institute and CNH	98 (41 families)	42	98	5	Rare monogenic diseases	P and A	NA
Hiatt et al. (2021)	United States of America	HudsonAlpha	6 (trio)	6	6	NA	NDDs	NA	2:4
Sinha et al. (2025)	UAE	Dubai Health Genomic Medicine Center	68	51	51	17	Rare diseases (45% neurological cases)	P and A	24:22 22 NA

lrWGS, long-read whole genome sequencing; F, female; M, male; P, pediatrics; A, adults; SVs, structural variants; CNH, Children’s National Hospital; RCIGM, Rady Children’s Institute for Genomic Medicine; GA4K, genomic answers for kids; AnDDI, anomalies of developmental delay and intellectual disability; KFSHRC, king faisal specialist hospital and research center; HudsonAlpha, HudsonAlpha Institute for Biotechnology, Huntsville; DEEs: developmental and epileptic encephalopathies; NDDs, neurodevelopmental disorders; NA, not available or not reported.

### 3.2 Quality of the eligible studies

**FIGURE 2:**
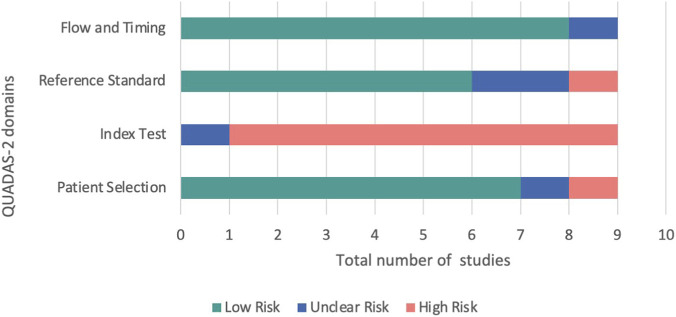
Risk of bias assessment using the QUADAS-2 tool.

The risk of bias across the nine studies was evaluated using the QUADAS-2 tool (Figure 2). Overall, most studies had a low risk of bias in the domains of patient selection, reference standard, and flow and timing. However, the index test domain was rated as high risk in all studies, primarily due to the lack of blinding to prior genetic test results. This reflects routine clinical practice where lrWGS is applied as a second-tier test rather than a standalone diagnostic tool.

### 3.3 Diagnostic yield of lrWGS

Among the nine included studies, a total of 646 previously unresolved or inconclusive cases underwent lrWGS. In the study by (AlAbdi et al., 2023), cases were reported by family rather than individuals; therefore, each family was counted as a single case, as only one affected individual per family was tested. Of the total cases, 29 were diagnosed exclusively through lrWGS, yielding an overall diagnostic rate of 4.5% (29/646). Diagnostic yield varied across studies depending on cohort size, selection criteria, and whether lrWGS was applied as a first-tier test or as a targeted follow-up to previous short-read testing. Most studies performed lrWGS after prior short-read WES/WGS or targeted gene panel sequencing. The platforms used included PacBio Sequel II/IIe and Oxford Nanopore PromethION. Detailed diagnostic yield and variant characteristics of diagnosed cases are summarized in Tables 3, 4.

**TABLE 3:** Primary outcome (diagnostic yield) of lrWGS in unresolved rare disorders cohorts from included studies.

Study	Prior genetic testing	LRS platform	Average coverage	Variant analysis targets	References genome	LRS-diagnosed cases/Tested (n/N)	LRS- diagnostic yield (%)	Comments
Cohen et al. (2022)	srWES, srWGS, or panel testing	PacBio Sequel II	≥ 30	SNVs, SVs, TRs	GRCh38	6/472	1.27	Diagnostic yield recalculated based on methods section and Supplementary Table S1, including only lrWGS-genotyped cases (n = 472) with prior negative testing and lrWGS-only solved cases. Original abstract yield (64/584) included mixed technologies
Colin et al. (2023)	Array-CGH, Sanger Sequencing, gene panel testing, srWES	ONT- PromethION	NA	SVs	GRCh37/hg19	1/2	-	Diagnostic yield was not calculated as LRS was performed on only two cases
De Clercq et al. (2024)	Karyotyping, FISH, microarray, shallow WGS, srWES, Mate-pair sequencing	ONT- MinION or PromethION	NA	SVs	GRCh38	2/6	-	Diagnostic yield not calculated as LRS was applied to a preselected cohort with unresolved but previously identified SVs
LaFlamme et al. (2024)	Gene panel, WES, or srWGS	ONT-PromethION	NA	Methylation	GRCh38	3/3	-	Diagnostic yield not calculated as LRS was selectively applied for DMR confirmation; only 3 cases underwent lrWGS.
Sanford Kobayashi et al. (2022)	srWGS	Pacbio Sequel II and IIe	25.2–38.8	SNVs, SVs, Methylation	GRCh38	1/30	3.33	Diagnostic yield recalculated as 3.33% (1/30) based on full LRS-tested cohort; original 25% (1/4) yield was subgroup-specific (immunodeficiency)
AlAbdi et al. (2023)	srWES	Pacbio Sequel IIe	NA	SNVs, SVs	GRCh37/hg19	6/34	17.65	Diagnostic yield recalculated as 17.65% (6/34) based on LRS-only diagnoses; original study reported 38% (13/34), including WES reanalysis
Negi et al. (2025)	srWGS	ONT-PromethION	36	SNVs, SVs, TRs, Methylation	GRCh38	3/42	7.14	Diagnostic yield recalculated as 7.14% (3/42) based on LRS-only diagnoses; the study reported 11/42, but remaining were identifiable by SRS reanalysis or from the positive cohort
Hiatt et al. (2021)	Array, Single gene test(s) or panel(s), srWES, srWGS	PacBio Sequel II	25–44	SNVs, SVs	GRCh38	2/6	33.33	Two LRS-solved cases reported; cohort selected based on absence of causal variants
Sinha et al. (2025)	srWES, microarray	ONT - PromethION	49	SNVs, SVs, CNVs, Methylation	GRCh37/hg19	5/51	9.80	Diagnostic yield (5/51) aligns with the study’s reported results

LRS, long-read sequencing; lrWGS, long-read whole genome sequencing; srWES, short-read whole exome sequencing; srWGS, short-read whole genome sequencing; SNVs, single nucleotide variants; SVs, structural variants; CNVs, copy number variants; TRs, tandem repeats; DMRs, differentially methylated regions; WES, whole exome sequencing; ONT, oxford nanopore technologies; PacBio, Pacific Biosciences; NA, not available or not reported; FISH, fluorescence *in situ* hybridization.

LRS-Diagnosed Cases/Tested (n/N), Number of cases diagnosed exclusively by lrWGS, shown as n (LRS-only diagnosed cases) out of N (total number of previously negative or unresolved cases tested by lrWGS). LRS, Diagnostic Yield (%), Percentage of cases uniquely solved by lrWGS, calculated as (n/N × 100%).

**TABLE 4:** Summary of diagnosed cases by lrWGS in included studies.

Study/Citation	Clinical phenotype	Gene (OMIM ID)	Variant type	Gene region	Zygosity	Final reported diagnosis	Comments/LRS contribution
Cohen et al. (2022)	Septo-optic dysplasia, hypotonia, strabismus, tremor, DD	*AARS2* (MIM: 612035)	SNV, SV (deletion)	SNV: Exon 4 SV: Exons 5–7	AR/compound het	AARS2-related disease	The SNV was detected previously; however, deletion was difficult to detect using prior genetic testing
Cohen et al. (2022)	Global DD, dystonia	*STARD7* (MIM: 616712)	TR (pentamer expansion)	Intron 1	AD/HET	Novel expansion disorder	​
Colin et al. (2023)	Alopecia, micropenis, hypotonia, global developmental delay, autism spectrum features, attention deficit, wide-based gait	*LSS* (MIM: 600909), *MCM3AP* (MIM: 603294)	SNV, SV (deletion)	SNV: Exonic SV: Promoter, exonic	AR/HOM	Consistent with OMIM; Alopecia-intellectual disability syndrome 4 (MIM: 618840)	The SNV was detected previously; however, SV was missed by SRS.
De Clercq et al. (2024)	Severe intellectual disability and dysmorphic facial features	*CELF2* (MIM: 602538)	SV (translocation)	Repeat region (chr9), coding region (chr10)	AD/HET	Consistent with OMIM; Developmental and epileptic encephalopathy (MIM: 619561)	Previously identified by karyotyping and confirmed by FISH. Breakpoints of the t (9; 10) translocation were precisely delineated using LRS, enabling a novel molecular diagnosis
De Clercq et al. (2024)	Mild intellectual disability, Down-like features, and autism spectrum disorder	*MYT1L* (MIM: 616521)	SV (deletion)	Exon 9	AD/HET	Consistent with OMIM; Intellectual developmental disorder, autosomal dominant (MIM: 616521)	Previously identified through WES. The exon 9 *MYT1L* deletion was precisely delineated using LRS.
LaFlamme et al. (2024)	Epilepsy of infancy with migrating focal seizures (EIMFS)	Multiple genes	Hypermethylation- SV (translocation)	Multiple genes	AD/HET	NA	DMR was detected; LRS was performed to confirm the underlying cause
LaFlamme et al. (2024)	Developmental and epileptic encephalopathies	*CSNK1E* (MIM: 600863)	Hypermethylation- CGG repeat	5′UTR and intron 1	AD/HET	NA	DMR was detected; LRS was performed to confirm the underlying cause
Sanford Kobayashi et al. (2022)	Hepatosplenomegaly; laboratory evaluation indicated abnormal immunoglobulins and imaging was remarkable for abnormal osseous mineralization	*IKBKG* (MIM: 300248)	SNV	Exon 10 (stop-loss variant)	X-linked	Immunodeficiency with or without anhidrotic ectodermal dysplasia	NGS dead zone due to a pseudogene with greater than 99% homology
AlAbdi et al. (2023)	Increased serum lactate, encephalopathy, abnormal breathing, metabolic acidosis, widened subarachnoid space, focal T2 hyperintense brainstem lesion	*TYMS* (MIM: 188350)	SV (insertion)	Intron 3	AR/HET	Lethal neonatal lactic acidosis	Novel phenotype
AlAbdi et al. (2023)	Hearing impairment, microcephaly, short stature, decreased body weight, Infra-orbital crease, capillary hemangiomas (flat), hypertonia, failure to thrive	*STK25* (MIM: 602255)	SV (deletion)	Deletion of regulatory region in *STK25* + deletion of intron 20 of *ANO7*	AR/HOM	STK25-related neurodevelopmental disorder	Novel phenotype
AlAbdi et al. (2023)	Leber congenital amaurosis	*RP1L1* (MIM: 608581)	SV	Exonic, in-frame	AR/Compound het	Consistent with OMIM; Retinitis pigmentosa 88 (OMIM: 618826)	Known gene and phenotype
AlAbdi et al. (2023)	Band keratopathy, glaucoma, corneal calcification, choroidal detachment	*SLC4A4* (MIM: 603345)	SNV	Promoter region	AR/HOM	*SLC4A4*-related band keratopathy	Novel phenotype
AlAbdi et al. (2023)	Dwarfism, microcephaly, global developmental delay, spastic tetraparesis, gastroesophageal reflux, failure to thrive, abnormal racial shape, deeply set eyes, prominent nose, hypoplasia of dental enamel, high palate, abnormal sternum morphology, brain atrophy (mild), interhemispheric cyst (small)	*SNAP91* (MIM: 607923)	SNV	Intronic	AR/HOM	*SNAP91*-related microcephalic primordial dwarfism	Novel phenotype
AlAbdi et al. (2023)	Microcephaly, plagiocephaly, abnormal number of hair whorls, anteverted nares, microtia, deep palmar crease, slow saccadic eye movements, heart murmur, distal arthrogryposis, adductor longus contractures, Increased muscle tone,	*LEMD2* (MIM: 616312)	Indel	Intron 4	AR/HOM	*LEMD2*- related neurodevelopmental disorder	Novel phenotype
​	global developmental delay, failure to thrive, diffuse white matter abnormalities, hip dysplasia dilatation of the renal pelvis, mildly echogenic liver	​	​	​	​	​	​
Negi et al. (2025)	NA	*CYP21A2* (MIM: 613815)	SNV/SV (deletion)	Intron/Exon	AR/Compound het	Consistent with OMIM; Adrenal hyperplasia, congenital, due to 21-hydroxylase deficiency (MIM: 201910)	Used Parakit, novel collapsed pangenome method for mapping long reads
Negi et al. (2025)	NA	*LHCGR* (MIM: 152790)	SNV/SV (deletion)	SNV: Exon 11 SV: Deletion of Exon 9	AR/Compound het	NA	Biallelic variants in LHCGR were detected using LRS-only. Both variants phased by LRS in absence of parental data
Negi et al. (2025)	Complex neurodevelopmental phenotype including minimal expressive language, autistic features, and dysmorphic features	*ARID1B* (MIM: 614556)	SNV/Methylation	Intronic, splicing effect	AD/HET	Consistent with OMIM; Coffin-Siris syndrome 1 (MIM: 135900)	Methylation information provided through LRS.
Hiatt et al. (2021)	Intellectual disability, developmental delay, and seizures	*CDKL5* (MIM: 300203)	SV (insertion)	Exon 3	XLD	Consistent with OMIM; Developmental and epileptic encephalopathy 2 (MIM: 300672)	Mosaicism is suspected according to SNV analysis near the insertion region
Hiatt et al. (2021)	Intellectual disability, facial dysmorphism, hypotonia	Multuple genes *DGKB* (MIM: 604070) *MLLT3* (MIM: 159558)	SV (insertion-translocation-inversion)	*MLLT3*: Exons 3–4, exons 9–10	AR/HET	​	Two insertional translocations between chromosomes 7 and 9 and an inversion result in disruption of two protein-coding genes. Expression of *DGKB* was not examined, as the gene is not expressed at appreciable levels in blood
Sinha et al. (2025)	Developmental delay, ID, dysmorphic features, obesity	*NSMCE3* (MIM: 608243)	SV (deletion)	Multiple regions	AD/HET	Consistent with OMIM; Lung disease, immunodeficiency, and chromosome breakage syndrome (MIM: 617241)	​
Sinha et al. (2025)	Eye disorder-foveal hypoplasia and/or anterior segment dysgenesis	*SLC38A8* (MIM: 615585)	SNV/SV (deletion)	Exons 8–3′UTR	Compound het	Consistent with OMIM; Foveal hypoplasia 2, with or without optic nerve misrouting and/or anterior segment dysgenesis (MIM: 609218)	​
Sinha et al. (2025)	Learning disabilities with distinctive brittle hair, a hallmark of trichothiodystrophy non- photosensitive 1 associated with non-functional MPLKIP protein	*MPLKIP* (MIM: 609188)	SV (deletion)	3′UTR	AR/HOM	Consistent with OMIM: Trichothiodystrophy 4, nonphotosensitive (MIM: 234050)	Further investigation is required to confirm this mechanism and to understand the functional impact of the 3′UTR deletion in this gene
Sinha et al. (2025)	Craniosynostosis, intellectual deficit, short stature, and facial dysmorphism	*NSD1* (MIM: 606681)	SV (duplication) and Methylation	Multiple regions	AD/HET	Consistent with OMIM; Sotos syndrome (MIM: 117550) Methylation: Hunter McAlpine syndrome (HMA)	​
Sinha et al. (2025)	Skeletal/musculoskeletal	SMN1 (MIM: 600354)	Methylation	NA	AR/HOM	Consistent with OMIM: Spinal muscular atrophy	Patient has 4 copies of *SMN2*

AD, autosomal dominant; AR, autosomal recessive; CNV, copy number variant; DD, developmental delay; DMR, differentially methylated region; FISH, fluorescence *in situ* hybridization; HET, heterozygous; HOM, homozygous; ID, intellectual disability; Indel, insertion/deletion; LRS, long-read sequencing; OMIM, online mendelian inheritance in man; SRS, short-read sequencing; SNV, single nucleotide variant; SV, structural variant; TR, tandem repeat; UTR, untranslated region; WES, whole exome sequencing; XLD, X-linked dominant.

### 3.4 Variant types and genomic features identified

**FIGURE 3:**
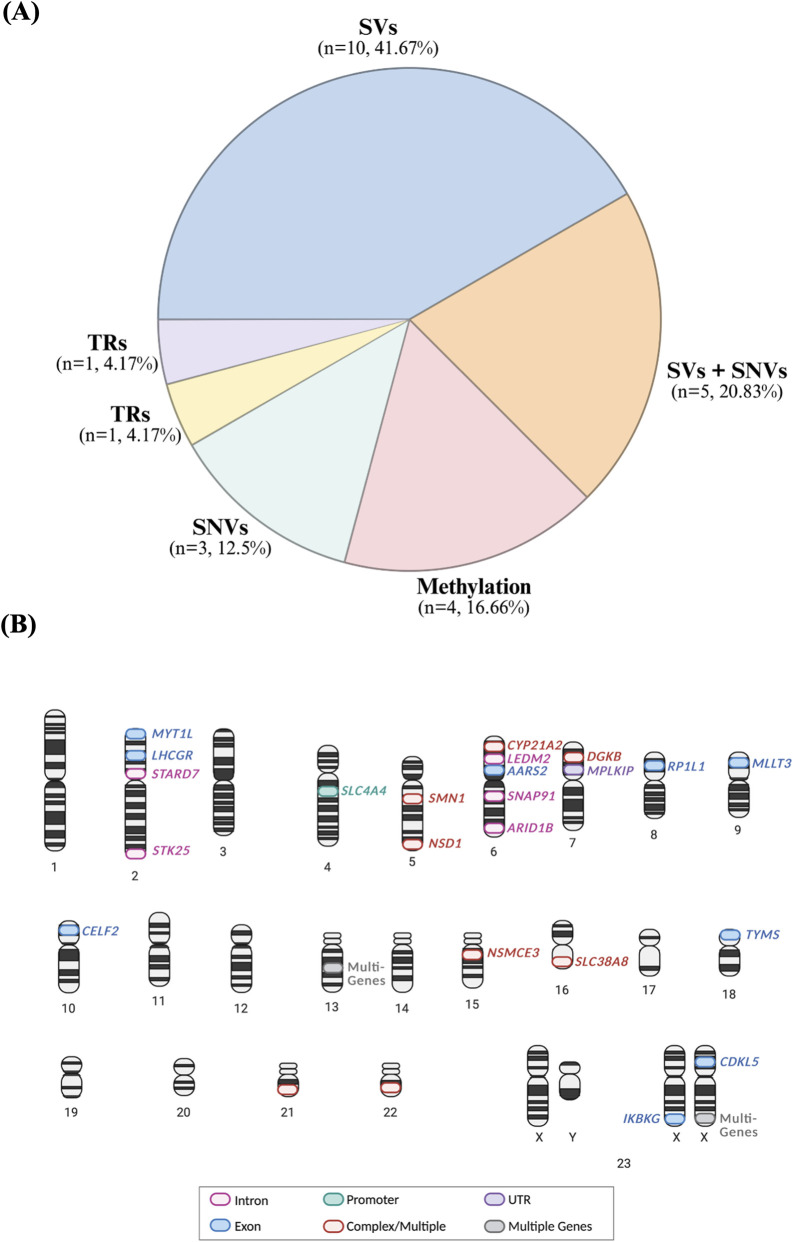
Genetic findings identified by lrWGS in cohorts with unresolved rare genetic disorders. **(A)** Pie chart showing the distribution of variant types identified through lrWGS. **(B)** Chromosomal map of affected genes based on lrWGS-detected variants. Genes are color-coded by the genomic region impacted (e.g., exon, intron, promoter, UTR, complex/multiple, or multi-gene). When a specific diagnostic gene was reported, it is shown; in cases where no single gene was specified due to complex rearrangements or broad regions, the label “Multi-Genes” is used. Detailed genomic coordinates and full gene lists are provided in the Supplementary Materials. Abbreviations: SNV, single nucleotide variant; SV, structural variant; CNV, copy number variant; TR, tandem repeat; UTR, untranslated region.

A total of 24 unique diagnoses were reported across 29 diagnosed cases involving 25 genes. The distribution of variant types detected by lrWGS is shown in Figure 3A. SVs were the most frequently identified, accounting for 41.67% of diagnostic findings (n = 10), followed by combined SV/SNV events in heterozygous inheritance (20.83%, n = 5), methylation profiling (16.67%, n = 4), SNVs (12.50%, n = 3), and other categories such as indels and tandem repeats (TRs), each contributing 4.17% (n = 1). Many of the detected variants missed using SRS were within noncoding regions (e.g., introns, promoters), or complex regions (e.g., insertions, translocations, methylation, and pseudogenes).

Chromosome-level mapping of the implicated genes is shown in Figure 3B, with affected genes color-coded based on the genomic region impacted. Where a specific diagnostic gene was reported, it is shown on the map; in cases involving complex rearrangements with no single implicated gene, the label “Multi-Genes” was used. A full list of coordinates and variant annotations is provided in the Supplementary Table.

## 4 Discussion

Despite significant advances in NGS, up to half of individuals with rare genetic disorders remain undiagnosed. One potential causative factor is the limitation of SRS-based methods in detecting complex genomic alterations such as SVs, repetitive regions, and epigenetic changes. This systematic review aimed to evaluate whether lrWGS provides added diagnostic value in patients with previously unresolved rare diseases. To our knowledge, this is the first comprehensive review focusing on the application of lrWGS in clinical cohorts that had negative or inconclusive results from prior genetic testing.

From an initial screen of 6,024 articles, a total of nine studies were finally included, comprising 646 individuals who had previously received inconclusive or negative results from standard genetic testing. Of these, 29 were newly diagnosed through lrWGS (24 unique diagnoses involving 25 genes), resulting in an overall diagnostic yield of 4.5%. While this diagnostic yield of 4.5% is clinically meaningful, particularly given that all included cases had undergone extensive prior testing, it remains modest. Several factors may contribute to this. First, lrWGS is still an emerging technology, and both laboratory workflows and bioinformatics tools are continuously evolving; for example, recent consensus-based frameworks that integrate multiple structural-variant callers have demonstrated improved accuracy and robustness, highlighting the importance of ongoing methodological development (Pietryga et al., 2025). Second, although lrWGS enables improved detection of variants in non-coding regions, including deep intronic and regulatory elements, the interpretation of such variants remains challenging due to incomplete functional genomic annotation and limited clinical frameworks for assessing pathogenicity (Austin-Tse et al., 2022). Third, using reference genomes such as GRCh38 can limit the detection of variants in highly repetitive or previously unresolved regions; new resources like the Telomere-to-Telomere (T2T-CHM13) reference genome have been shown to improve read mapping accuracy and variant detection in these regions, thereby boosting diagnostic performance (Aganezov et al., 2022). However, their clinical use remains limited because annotation tools and databases still mainly depend on GRCh38. Finally, emerging artificial intelligence–based methods for genome interpretation, such as AlphaGenome, are showing promise in enhancing the prioritization and functional prediction of non-coding variants, especially in regulatory and deep intronic regions; however, their clinical use remains in the early stages (Avsec et al., 2026). Together, these factors suggest that the current diagnostic yield likely underestimates the full potential of lrWGS as both technologies and analytical frameworks continue to develop.

The diagnostic yield varied across the included nine studies, likely due to several factors such as sample size, patient selection criteria, variability in alignment and variant calling tools, disease focus, sequencing platform, extent of prior genetic testing, and the specific application of lrWGS (whether used as a follow-up to confirm suspected findings or as a first-tier comprehensive standalone analysis). The investigative focus (e.g., SVs, SNVs, methylation profiling) also influenced yield.

The highest diagnostic yield was reported in (Hiatt et al., 2021), at 33.3% (2 of six individuals) using the PacBio sequencing platform. However, due to the small sample size and the specific focus on neurodevelopmental disorders, the generalizability of this result is limited. Among larger cohorts, (AlAbdi et al., 2023), reported a diagnostic yield of 17.65% (6 out of 34 families), also using PacBio. All participants had undergone WES prior to lrWGS, and the higher yield can be partially attributed to the fact that applying lrWGS provided insights into intronic and regulatory regions that were previously undetectable using WES. Notably, five of the six solved cases involved non-coding variants in genes such as *TYMS*, *STK25*, *SLC4A4*, *SNAP91*, and *LEMD2* (see Supplementary Material for details). A comparable yield was reported in (Sinha et al., 2025) at 9.8% (5 out of 51) using ONT. Like (AlAbdi et al., 2023), this study applied lrWGS after WES and included methylation analysis, which contributed to the identification of disease-causing variants. The cohort was predominantly of Arab ancestry and included a high proportion of neurological cases, further supporting the value of lrWGS in diverse and clinically complex populations. In (Negi et al., 2025), ONT was used following prior WGS, yielding a 7.1% diagnostic rate (3 out of 42). This study integrated SNV, SV, and methylation analyses and employed Parakit, a pangenome assembly-based method, enabling resolution of complex regions such as *CYP21A2*. Additionally, phasing allowed identification of compound heterozygous variants in *LHCGR* despite the absence of parental data. Sanford Kobayashi et al. (Sanford Kobayashi et al., 2022) applied PacBio to 30 previously undiagnosed cases and reported a 3.3% diagnostic yield (1 case). Although the original study reported a higher percentage (25%), that number was based on a small disease-specific subgroup. When recalculated across the full lrWGS-tested cohort, the more conservative estimate more accurately reflects the yield attributable solely to lrWGS (Sanford Kobayashi et al., 2022).

The lowest diagnostic yield was seen in (Cohen et al., 2022), where PacBio was used on a large cohort of 472 individuals who had undergone a range of prior testing methods (WES, WGS, gene panels). Despite this, lrWGS uniquely identified diagnoses in six individuals (1.3%), which, though modest, demonstrates incremental value in highly pre-screened populations.

Several studies applied lrWGS selectively and did not permit diagnostic yield calculation. For instance, (Colin et al., 2023), performed ONT sequencing on only two cases from a cohort of 15 unresolved Mendelian disorders, identifying a diagnosis in one. Due to the limited testing, yield was not calculated. Similarly, (De Clercq et al., 2024), applied lrWGS to a small, preselected group of six individuals with previously identified but unresolved SVs. While two were solved, the pre-enrichment bias prevents generalization. (LaFlamme et al., 2024). used ONT to investigate methylation in three individuals with suspected imprinting disorders and successfully confirmed differentially methylated regions (DMRs) in all three; however, since lrWGS was applied only to confirm prior findings, the yield was not assessed.

With respect to the genetic findings in cases exclusively solved by lrWGS, SVs represented the largest category, accounting for 41.67% (10 out of 24 cases). These were followed by combined SV/SNV findings in a compound heterozygous state (20.83%, five out of 24), methylation-related alterations (16.66%, four out of 24), SNVs (12.50%, three out of 24), TRs, and indels, each contributing 4.17% (1 out of 24). These categories highlight the well-documented limitations of SRS and the strengths of long-read platforms, which can span repetitive and GC-rich regions, resolve complex SV breakpoints, and detect non-sequence-based alterations such as differential methylation (Romagnoli et al., 2023; Wojcik et al., 2023). The affected genomic regions were diverse, with variants identified in introns, promoters, UTRs, exons, and even across multiple genes. Many of these regions are typically inaccessible or poorly covered by short-read methods, particularly WES. Notably, some diagnoses involved highly complex rearrangements such as insertion-translocation-inversion events spanning multiple genes, including *DGKB* and *MLLT3*. While PacBio HiFi reads were generally preferred for high-accuracy SNV and SV detection, ONT was frequently used in studies investigating methylation profiles or requiring rapid turnaround due to its real-time sequencing capabilities (LaFlamme et al., 2024; Negi et al., 2025; Olivucci et al., 2024; Sinha et al., 2025; Zheng et al., 2023).

Interestingly, the diagnostic gains from lrWGS were not only technical but also interpretative. In several cases, prior SRS had already flagged one pathogenic variant, but phase determination or confirmation of compound heterozygosity was not possible without parental samples. LRS enabled phasing in such situations, successfully resolving compound heterozygosity in genes like *LHCGR* and *CYP21A2* (Negi et al., 2025). Moreover, lrWGS provided novel clinical insights. Some cases involved potentially new gene-disease associations, such as *TYMS*-related lactic acidosis, while others revealed atypical presentations of known disorders, including *STK25*-associated neurodevelopmental delay. These examples highlight the ability of long-read sequencing not only to close diagnostic gaps but also to expand phenotype-genotype correlations and uncover new disease mechanisms (AlAbdi et al., 2023).

Across the studies included in our systematic review, lrWGS was most often used as a second-tier diagnostic tool, following inconclusive SRS, and when no specific genetic diagnosis was suspected. However, emerging evidence suggests that phenotype-guided LRS of known disease genes, for example, *GBA* in Parkinson’s disease (Graham et al., 2020), *PKD1* in autosomal dominant polycystic kidney disease (ADPKD) (Hort et al., 2023), and *MUC1* in autosomal dominant tubulointerstitial kidney disease (Okada et al., 2022), can result in substantially higher diagnostic yields, reaching 50%–70%, particularly when guided by strong clinical suspicion.

Notably, ONT enables real-time analysis, which may support rapid sequencing in specific clinical contexts, and has been explored as a potential first-tier diagnostic tool in critically ill pediatric patients (Kamolvisit et al., 2025). Additional advantages include the simultaneous detection of methylation and sequence variants in a single assay and its capacity to phase alleles even in the absence of parental samples. However, several challenges currently limit its broader clinical implementation. These include higher base-level error rates than short-read sequencing, a lack of standardized bioinformatics pipelines, and ongoing difficulties in interpreting structural and non-coding variants. In addition, higher costs, lower throughput, and increased computational and data storage requirements pose practical barriers to routine use (Amarasinghe et al., 2020). While the overall improvement in diagnostic yield remains modest, the available evidence suggests that, when applied in targeted, phenotype-driven scenarios, long-read genome sequencing holds meaningful clinical utility and continues to contribute to the discovery of novel and complex genetic diagnoses.

Despite these promising results, our review has several limitations. Many of the included studies lacked consistency in prior genetic testing approaches, complicating comparisons of diagnostic yield. Sample sizes were generally small, and some cohorts were subject to selection bias. As reflected by the QUADAS-2 assessment, all studies had a high risk of bias in the index test domain, primarily due to a lack of blinding. Furthermore, most studies originated from North America and Europe, with only two studies from the Middle East (AlAbdi et al., 2023; Sinha et al., 2025) emphasizing the need for broader geographic and ancestral representation in future research.

## 5 Conclusion

Our findings highlight the emerging role of lrWGS in diagnosing previously unresolved rare genetic disorders, especially when applied after WES and combined with phasing and methylation profiling. While the overall diagnostic yield remains modest, lrWGS demonstrates clear advantages in detecting complex and previously inaccessible variants.

As sequencing technologies continue to evolve, we anticipate reductions in cost, improvements in accuracy, and the development of more targeted long-read applications, including panels or exome-level tests. With its real-time analysis, ability to phase alleles, and single-test methylation detection, lrWGS may soon find broader utility as a first-line genetic tool for both diagnostic and screening applications.

Future research could focus on expanding population diversity, standardizing prior testing protocols, and reducing bias through blinded (un-selected) assessment. With continued innovation and clinical integration, long-read genome sequencing has the potential to significantly enhance diagnostic outcomes and further our understanding of rare genetic diseases globally.

## Data Availability

The original contributions presented in the study are included in the article/Supplementary Material, further inquiries can be directed to the corresponding author.
